# Prognosis of lymph node-negative breast cancer: Association with clinicopathological factors and tumor associated gene expression

**DOI:** 10.3892/ol.2014.2339

**Published:** 2014-07-10

**Authors:** JING HE, HAIJUAN WANG, FEI MA, FENGYI FENG, CHEN LIN, HAILI QIAN

**Affiliations:** 1 Department of Internal Medicine, Cancer Institute and Hospital, Chinese Academy of Medical Sciences, Beijing 100021, P.R. China; 2 State Key Laboratory of Molecular Oncology, Cancer Institute and Hospital, Chinese Academy of Medical Sciences, Beijing 100021, P.R. China

**Keywords:** lymph node-negative breast cancer, prognosis, HER2, TOP2A, CCND1

## Abstract

The aim of the present study was to investigate the association between the prognosis of lymph node-negative breast cancer patients and clinicopathological factors, as well as the association between tumor-associated gene expression and prognosis. Clinical data and survival information was collected for 341 patients with lymph node-negative breast cancer, admitted to the Cancer Hospital of the Chinese Academy of Medical Sciences (Beijing, China) from 1995 to 1999. Kaplan-Meier survival analysis and Log-rank tests were used to evaluate the association of clinical parameters and prognosis. In addition, the gene expression of HER2, TOP2A and CCND1 in patients with good [disease-free survival (DFS), ≥5 years] and poor (DFS, <5 years) prognoses was analyzed. The clinicopathological factors of the 341 lymph node-negative breast cancer patients were determined. The 5-year DFS and overall survival rate (OS) in patients >35 years old was higher as compared with those of patients under the age of 35. Tumor size significantly affected the 5-year DFS. Patients with smaller tumors (≤2 cm) had a significantly higher DFS rate as compared with patients with larger tumors (>2 cm). Estrogen receptor (ER)-positive patients had a significantly higher 5-year DFS and OS rate as compared with ER-negative patients. By contrast, there were no significant differences in the 5-year DFS and OS rates between progesterone receptor-positive and -negative patients. The 5-year DFS and OS rates were significantly higher in patients treated with adjuvant hormone therapy, as compared with patients without hormone therapy. The expression of HER2 protein was higher in patients with a poor prognosis as compared with those with a good prognosis; however, there were no differences in the protein expression of CCND1 and TOP2A between patients with a good and poor prognosis. The results of quantitative polymerase chain reaction showed that the gene expression of HER2 and CCND1 was higher in patients with a poor prognosis as compared with that in patients with a good prognosis. TOP2A gene expression was not significantly different between patients with a poor and good prognosis. The age at diagnosis, tumor size, ER status and hormone therapy were associated with prognosis in patients with lymph node-negative breast cancer. The molecular biomarker, HER2, but not CCND1 or TOP2A, may be a critical factor for predicting prognosis.

## Introduction

Over the last two decades, breast cancer has remained the most common type of cancer in females in China (1). The incidence of breast cancer has significantly increased, but that of breast cancer-associated mortality is decreasing over time due to the development of new diagnostic approaches, the release of new drugs and the understanding of the molecular pathology of this disease (2). This has encouraged research on novel and more efficient treatments that overcome the limitations of conventional chemotherapy. Although adjuvant therapy results in a survival advantage, the toxicity associated with these therapies is significant. Thus, the balance between the risks, costs and the potential benefits for breast cancer patients is of importance (3). Patients with lymph node-negative breast cancer exhibit good biological behaviors, but how these patients will be benefit from cytotoxicity treatments is unknown. Therefore, it is important to evaluate the association between prognosis and clinical pathological factors, and to establish an appropriate treatment regimen.

The potential factors affecting the prognosis of lymph node-negative breast cancer include tumor diameters (<1 cm) and the status of hormone receptors (4). The prognosis for lymph node-negative females with small tumors is very good, with a 5-year disease free survival (DFS) rate of 100%, which decreases with an increase in tumor size (5). Hormone receptors, such as estrogen receptor (ER) and progesterone receptor (PR), are considered not only prognostic factors, but also as biomarkers to evaluate the efficiency of adjuvant therapy (6). The survival rate of ER-positive patients is higher as compared with that of ER-negative patients (7). Other factors associated with lymph node-negative breast cancer include the age at which the disease is diagnosed, tissue type of tumor and classification (8). The identification of additional prognostic factors will assist physicians in determining the appropriate therapeutic approach to follow.

Overexpression of cancer-related genes is characteristic of cancer cells and allows overproduction of proteins responsible for the acquisition and maintenance of malignant phenotypes (9). Oncogenes function in the progression of breast cancer (10,11). Previous studies have shown that HER2, an epidermal growth factor (EGF), is detected in ~25% breast cancer patients, and is associated with a poor prognosis (12,13). However, the association between HER2 and lymph node-negative breast cancer is controversial. Other potential genes, such as TOP2A and CCND1, have been suggested (9,14), but their association with lymph node-negative breast cancer are in disagreement. Enhanced understanding of the prognostic implication of oncogenes in patients with lymph node-negative breast cancer will provide more accurate prognostic information, and may influence the treatment options followed.

The present study aimed to investigate the association between clinicopathological factors and prognosis, as well as the association between tumor-related gene expression and prognosis for 341 patients with lymph node-negative breast cancer.

## Patients and methods

### Study population

The subjects of the present study included a cohort of 341 patients with lymph node-negative breast cancer from a total of 1347 breast cancer patients admitted to the Cancer Hospital of the Chinese Academy of Medical Sciences (Beijing, China) from 1995 to 1999. All 341 patients were treated with surgery in the early stages of cancer, and were followed up until 2005. Clinicopathological factors, including the age at which the diagnosis was made, menopausal status, tumor diameter, lymph node dissection, histopathological type, and ER and PR status, were collected. The 43 patients who exhibited recurrence were considered as the poor prognosis group, and 40/268 surviving patients were considered as the good prognosis group for gene and protein expression analysis. A total of 3/43 cases with poor prognosis were excluded as they only received a modified radical mastectomy. A total of 43 cases of breast fibroadenoma tissue were used as controls. The study was approved by the ethics committee of the Chinese Academy of Medical Sciences Cancer Hospital (no. NCC2013-038; Beijing, China).

### Immunohistochemistry (IHC)

Normal and tumor tissues were embedded in paraffin (35×27 mm), and the subsequent paraffin slices were observed under a microscope [BX46; Olympus (China) Co., Ltd., Beijing, China] to ensure that the samples had ≥50% of the tumor tissue. A tissue array was constructed using a tissue microarrayer (ATA-27; Beecher Instruments, Inc., Sun Prairie, WI, USA). The slides were stained by immunohistochemical methods, as previously described (15). The monoclonal mouse anti-human HER2 antibody (DakoCytomation, Glostrup, Denmark) was diluted 1:150 in phosphate-buffered saline (PBS). The monoclonal rabbit anti-human CCND1 and monoclonal mouse anti-human TOP2A primary antibodies were purchased from Zhongshan Biotech Co., Ltd., (ZA-0101; Zhongshan, China) and Fuzhou Maixin Biotech Co., Ltd (MAB-0588; Fuzhou, China), respectively. Control sections were incubated with PBS instead of the primary antibody as a negative control in each set of slides stained. The biotinylated polyclonal goat anti-mouse/rabbit IgG (ZB-2305, ZB-2301; Zhongshan Golden Bridge Biotechnology Co., Ltd., Beijing, China) was used as secondary antibody with a 1:2,000 dilution. Following streptavidin-biotinylated horseradish peroxidase complex incubation, the slides were stained with 3,3′-diaminobenzidine. The slides were then counterstained with hematoxylin, and mounted with neutral balsam. HER2 was stained brown in the cell membrane.

For data analysis, the Hercep Test Score method was used as follows (15): Membrane staining in <10% of tumor cells was defined as 0; weak and incomplete membrane staining in >10% of cells was defined as 1^+^; moderate and complete membrane staining in >10% of tumor cells was defined as 2^+^; strong and complete membrane staining in >10% of tumor cells was defined as 3^+^. Samples with a score of 0 or 1^+^ were considered negative, and samples with a score of 2^+^ or 3^+^ were considered positive. Cells were considered positive for CCND1 and TOP2A staining when brown particles were observed on the nuclei. The percentage of positive cells in a slice was calculated. Positive staining was defined at three levels: 10–20% was considered as 1^+^, 20–50% was considered as 2^+^, and >50% was considered as 3^+^.

### HER2, CCND1, and TOP2A DNA expression

DNA was extracted from paraffin-embedded samples by dewaxing, hydration and digestion. The digestion buffer comprising comprising 50 mmol/l Tris HCl, 1 mmol/l EDTA, 0.5% Tween 20 and 1 mg/ml proteinase K was purchased from Millipore (#39450-01-6; Billerica, MA, USA). Tissues were centrifuged at 13,000 × g for 3 min after being immersed in dimethylbenzene overnight, and the supernatant was discarded (repeated for three cycles). The samples were then hydrated in sequential concentrations of ethanol (100, 95 and 70%). Digestion was performed with four to five volumes of digestion buffer (50 mmol/l Tris-HCl, 1 mmol/l EDTA, 0.5% Tween 20 and 1 mg/ml proteinase K) and incubated in a water bath for 48 h at 56°C, followed by 95°C for 8–10 min. The supernatant was collected following centrifugation at 13,000 × g for 3 min and stored at −20°C for polymerase chain reaction (PCR) analysis. Quantitative PCR (qPCR) was performed using an ABI Prism 7300 system (Life Technologies, Grand Island, NY, USA). The master mix included 12.5 μl SYBR^®^ Premix Ex Taq™ (Takara Bio, Inc., Shiga, Japan), 0.5 μl forward primer, 0.5 μl reverse primer, 1 μl DNA and 10.5 μl ddH_2_O. The PCR primer sequences were as follows: *Her2-*F,5′-GAACTGGTG TATGCAGATTGC-3′; *Her2-*R, 5′-AGCAAGAGTCCCCAT CCTA-3′. *Ccnd1-*F, 5′-GGGCAGTTTTCTAATGGAATGG-3′; *Ccnd1-*R, 5′-CACCACAGTGGCCCACACT-3′. *Top2a-*F: 5′-GCCAGAATCTGTTCGGTTCAAC-3′; *Top2a-*R: 5′-AGG AAACTGAGTGCCGGCTT-3′. GAPDH-F, 5′-CCCCA CACACATGCACTTAC-3′; GAPDH-R, 5′-CCTAGTCC CAGGGCTTTGAT-3′.

The samples were run in triplicate. The PCR conditions were as follows: Predenaturation for 10 sec at 95°C, 45 cycles of 95°C for 5 sec, 56°C for 31 sec for HER2 and 60°C for 31 sec for CCND1 and TOP2A, with an added dissociation stage. The relative gene expression was calculated relative to GAPDH according to the following equations:

- ΔCt = Ct (Target gene) − Ct (GAPDH)
- ΔΔCt = ΔCt (samples) − ΔCt (adjust samples)
- Gene expression = 2^−ΔΔCt^

2^−ΔΔCt^ ≥3 was considered to indicate gene overexpression, and 2^−ΔΔCt^ <3 was not considered to indicate overexpression.

### Statistical analysis

All statistical analyses were performed using SPSS, version 10.0 (SPSS, Inc., Chicago, IL, USA). The survival rate was analyzed using the Kaplan-Meier method, and the correlation between the clinicopathological factors and prognosis were performed by log-rank test. A χ^2^ test was used to determine whether there were significant differences in the mRNA and protein expression of HER2, CCND1 and TOP2A between good and poor prognosis patients. P<0.05 was considered to indicate a statistically significant difference.

## Results

### Clinicopathological factors and patient survival rate, and their association with prognosis

The 341 patients with lymph node-negative breast cancer were diagnosed between the ages of 18 and 82 years. Among them, 57.5% were premenopausal and 18.5% had a family history of tumors. The tumor diameters ranged from 0.5 to 8 cm, the median value was 2 cm and the average diameter was 2.6 cm. Among these 341 patients, 50% had small tumor (≤2 cm in diameter), and the tumors were located in the upper outer quadrant of the breast. According to the classification of the tumor in its pathology, 62.2% were simplex carcinomas, 25.2% were breast invasive ductal carcinomas and ~81.5% of the patients had more than 10 lymph node dissections (Table I).

Approximately 50% of patients received adjuvant chemotherapy, including cyclophosphamide, cisplatin, vincristine, methotrexate, fluorouracil, epirubicin, adriamycin and pirarubicin; while the remaining ~50% of patients received cyclophosphamide methotrexate fluorouracil. The proportion of patients that received radiotherapy and hormone therapies were 52.5 and 54.9%, respectively.

The 5-year DFS and overall survival (OS) rate in patients >35 years was 85.1 and 95.1%, respectively, which was significantly higher as compared with patients <35 years (DFS, P=0.01; OS, P=0.07). The diameter of the tumor significantly affected the 5-year DFS rate, and patients with small tumors (≤2 cm in diameter) had significantly higher DFS rates as compared with patients with large tumors (P=0.02). However, the diameter of the tumor had no significant effect on the OS rate (P=0.1). Patients who were ER-positive had a significantly higher 5-year DFS (P=0.006) and OS rate (P=0.0009) as compared with ER-negative patients. By contrast, there was no significant difference in the 5-year DFS (P=0.1) or OS rate (P=0.09) between PR-positive and -negative patients (Table II).

Overall, the patients who received hormone therapy in an adjuvant setting exhibited a significant improvement in both the mean DFS (P=0.003) and mean OS (P=0.002), as compared with those who did not receive hormone therapy. Further analysis indicated that patients who were premenopausal, had a large tumor (>2 cm) or were ER-positive were most likely to benefit from hormone therapy, as compared with patients who were postmenopausal, had a small tumor (≤2 cm) or were ER-negative (Table III).

### HER2, CCND1 and TOP2A protein expression

**Figure 1:**
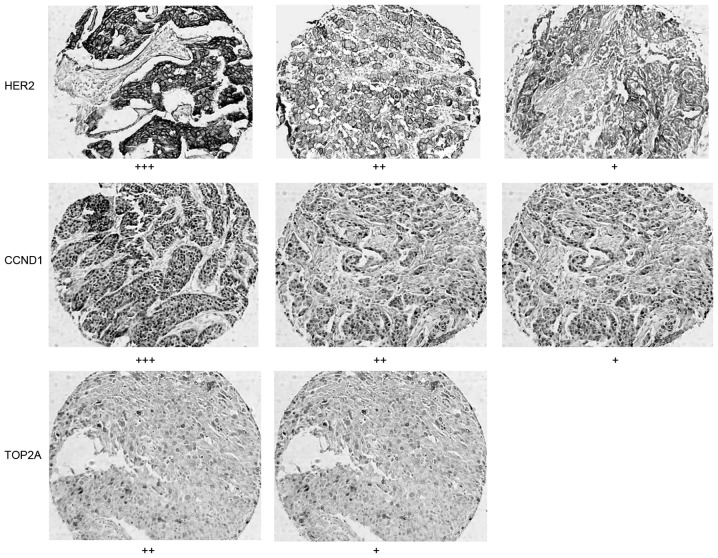
Immunohistochemistry of HER2, CCND1 and TOP2A in patients with lymph node negative breast cancer with a good and poor prognosis. Imaging was performed at ×200 magnification. HER2^+^, weak and incomplete membrane staining in >10% of cells; HER2^++^, moderate and complete membrane staining in >10% of tumor cells; HER2^+++^, strong and complete membrane staining in >10% of tumor cells. CCND1^+^ and TOP2A^+^, positive cells were 10–20%; CCND1^+^ and TOP2A^++^, positive cells were 20–50% ; CCND1^+^ and TOP2A^+++^, positive cells were >50%.

**Figure 2:**
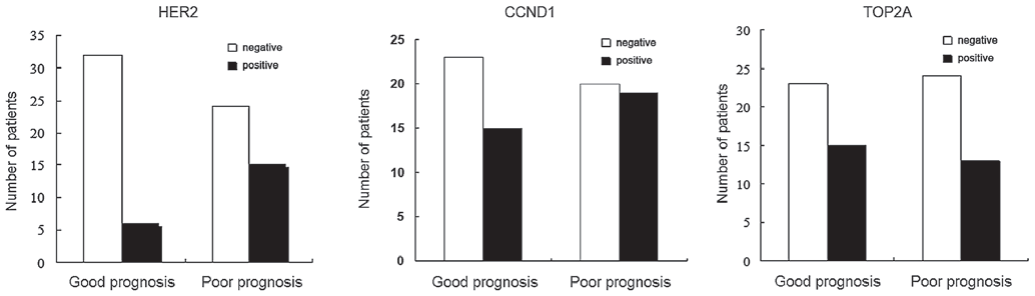
Protein expression of HER2, CCND1 and TOP2A in patients with lymph node-negative breast cancer, with a good and poor prognosis.

HER2 was stained brown in the cell membrane, whereas CCND1 and TOP2A were detected in the cell nuclei (Fig 1). Staining was performed on 77 cases of breast tumors, including 38 cases with a good prognosis and 39 cases with a poor prognosis. In addition, 43 cases of normal breast tissue were stained successfully in the prepared tissue array. IHC results showed that 27.2% (21/77) of patients with breast cancer expressed HER2, while no HER2 expression was detected in normal breast tissues. HER2 expression was detected in 15.8% (6/38) of the patients with a good prognosis, which was significantly lower than that in patients with a poor prognosis (38.5%, 15/39) (P=0.04). A total of 34/77 patients (44.2%) exhibited positive CCND1 protein expression. CCND1 expression in normal tissue was detected in only one case. There was no significant difference in the CCND1 protein expression between patients with a good (39.5%, 15/38) or poor (48.7%, 19/39) prognosis (P=0.5). TOP2A protein expression was detected in 36.4% (28/77) of tumor tissues, which was significantly higher as compared with that observed in normal tissues. However, there was no significant difference in TOP2A protein expression between patients with a good (39.5%, 15/38) or poor (33.3%, 13/39) prognosis (P=0.6) (Fig. 2).

### HER2, CCND1 and TOP2A gene expression

**Figure 3:**
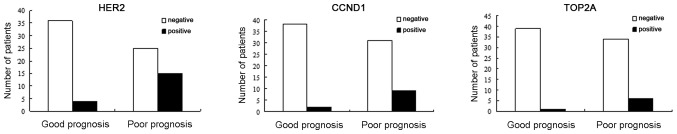
Gene expression of HER2, CCND1 and TOP2A in patients with lymph node-negative breast cancer, with a good and poor prognosis.

HER2 was expressed in 23.75% of the 80 patients with lymph node-negative breast cancer, in 37.5% (15/40) of patients in the poor prognosis group and in 10% (4/40) of patients in the good prognosis group. HER2 gene expression was detected at a higher frequency in the poor prognosis group as compared with the good prognosis group. The expression of CCND1 was significantly different between the good (22.5%, 9/40) and poor (5%, 2/40) prognosis groups (P=0.048). TOP2A expression was detected in 7/80 patients (8.75%). There was no significant difference in TOP2A expression between the good (2.5%, 1/40) and poor (15%, 6/40) prognosis groups (P=0.108) (Fig. 3). HER2, CCND1 and TOP2A gene expression were not associated with diagnosis age, menopausal status, tumor diameter or ER status.

## Discussion

The present study investigated the association between clinicopathological factors and survival rate in 341 patients with lymph node-negative breast cancer. To the best of our knowledge, the present study is the first to report that the expression of HER2, but not CCND1 or TOP2A, may be a critical predictor of a poor prognosis in the Chinese patients with lymph node-negative breast cancer.

In western countries, the majority of patients are diagnosed with lymph node-negative breast cancer after the age of 35, and only 4% of the patients are diagnosed with lymph node-negative breast cancer before the age of 35 (16). In Asian countries, lymph node-negative breast cancer is diagnosed at younger ages. The percentage of patients who are diagnosed with lymph node-negative breast cancer before the age of 35 has been reported to be 11.5% in the South Korean population (17), and 8.9% in the Chinese population (18). In the present study, it was identified that 9.4% of the patients with lymph node-negative breast cancer were diagnosed under 35 years old, which was consistent with the results of a previous study (18). In concordance with a study by Chung *et al* (19), the DFS rate was lower in patients <35 years as compared with patients >35 years, which implied that the younger age when lymph node-negative breast cancer was diagnosed, the worse prognosis. In a separate study, however, no clear association was identified between the age of diagnosis and prognosis (20). This disagreement may result from sample size, standardization and the range of age variations. It has been well documented that tumor size is a good predicator for prognosis, and it was considered that patients with a tumor diameter >2 cm were considered as at high risk of tumor recurrence (4). The 5-year OS of lymph node-negative breast cancer patients with small tumors (1 cm in diameter) has been reported as ~100%, and 75% of patients had no tumor recurrence or metastases 30 years following the initial diagnosis (5). In the present study, it was found that tumor size was associated with DFS, and patients with large tumors had a lower DFS and poor prognosis. No association was identified between tumor size and OS, which may be caused by the small population size and the relatively short follow-up period of the present study. Furthermore, it was identified that ER-positive patients exhibited a relatively longer DFS and OS as compared with ER-negative patients. This suggested that ER status was associated with prognosis, which was in agreement with previous results (21). ER status is a considered a good predictor for hormone therapy, and the death rate of ER-positive patients has been reduced by 5.6% after 5 years of hormone therapy (6). In the present study, there was no significant association between lymph node dissection and prognosis. A previous study, however, showed that patients with <10 lymph node dissections had a low DFS (22). The discrepancy of lymph node dissection and prognosis requires further investigation.

In 1998, the Early Breast Cancer Trialist’s Collaborative Group performed a meta-analysis of 30,000 cancer patients who received chemotherapy, and found that the mortality rate of patients <50 years old was reduced by 7%, and the mortality rate of patients between the ages of 51 and 69 years old was reduced by 2% (23). A similar report from the National Surgical Adjuvant Breast and Bowel Project showed that adjuvant chemotherapy improved the DFS and OS in lymph node-negative breast cancer and ER-negative patients (24). Chemotherapy, however, was not beneficial to patients with lymph node-negative breast cancer in the present study, which may be explained by the short period of chemotherapy and the limitation of drug application. By contrast, adjuvant hormone therapy improved the survival rate in the patients of the present study, which was reflected by the extension of the DFS and OS rate. Further analysis indicated that adjuvant hormone therapy had a combined effect on ER-positive and premenopausal patients, which was consistent with the results of previous studies (6,24,25). Hormone therapy was not observed to improve the survival status in postmenopausal patients; 81.4% of the postmenopausal patients in the present study had small tumors (tumor diameter, ≤2 cm), and all of these patients had a good prognosis. Differences in survival status were not apparent during the relatively short period.

The HER2 gene is located on human chromosome 17q12.1-q12.2. It encodes a 185-kDa transmembrane protein that belongs to the family of epidermal growth factor receptors. Approximately 30% of breast cancer primary lymph node-positive patients have been reported to exhibit HER2 overexpression (26), and ~60% of *in situ* carcinoma patients also have HER2 gene overexpression. Therefore, HER2 may be an early predictor of breast cancer (27). Ross *et al* (2003) demonstrated that HER2 overexpression was associated with poor prognosis in lymph node-negative breast cancer patients (28). In the present study, HER2 gene expression was analyzed in Chinese patients with lymph node-negative breast cancer. High HER2 gene and protein levels were shown to be associated with poor prognosis. In contrast to HER2, the association between TOP2A expression and lymph node-negative breast cancer patients was poor. As a critical protein in the regulation of DNA replication, TOP2A may be associated with the prognosis of lymph node-negative breast cancer (29). Previous studies, however, have suggested that there is no correlation between TOP2A expression and lymph node-negative breast cancer (30,31).

CCND1 is another potential molecular biomarker as a predictor of cancer prognosis. The CCND1 gene encodes cyclinD1, which is an initiation factor controlling G1 to S phase cell cycle transition. The CCND1 gene is located on human chromosome 11q13 and has been associated with numerous types of cancer, and has been shown to be expressed in 5–23% of breast cancer patients (32). CCND1 gene expression is also associated with estrogen and progesterone (33,34), but there has been disagreement concerning the association between CCND1 and prognosis (35,36), although CCND1 is expressed in ER-positive patients. In the present study, there was no association between CCND1 protein expression and prognosis in lymph node-negative breast cancer, but CCND1 gene expression was associated with poor prognosis. There have been other reports of inconsistent results between gene and protein expression in several other types of cancer (37,38). Possible reasons for a discrepancy include complex gene recombination, posttranscriptional control and protein translation.

In conclusion, the age at diagnosis, tumor diameter, ER status and hormone therapy increased the DFS and OS rate in Chinese patients with lymph node-negative breast cancer. Molecular biomarker HER2, but not CCND1 and TOP2A, may be a critical factor as a predictor of breast cancer prognosis.

## Figures and Tables

**Table I:** Clinicopathological factors of 341 lymph node-negative breast cancer patients.

	Patients, n (%)	Recurrence patients, n (%)	No disease survival, n (%)
Age of Diagnosis, years			
≤35	32 (9.4)	11 (15.1)	21 (7.8)
36–59	244 (71.6)	53 (72.6)	191 (71.3)
≥ 60	65 (19.1)	9 (12.3)	56 (20.9)
Menopausal status			
Premenopausal	196 (57.5)	47 (64.4)	149 (55.6)
Postmenopausal	145 (42.5)	26 (35.6)	119 (44.4)
Tumor diameter, cm			
≤2	176 (51.6)	29 (39.7)	147 (55)
2–5	142 (41.6)	39 (53.4)	103 (38.3)
≥5	23 (6.7)	5 (6.8)	18 (6.7)
Tumor site			
Upper out	160 (46.9)	37 (50.7)	123 (45.9)
Upper in	29 (8.5)	6 (8.2)	23 (8.6)
Bottom out	18 (5.3)	4 (5.4)	14 (5.3)
Bottom in	82 (24)	15 (20.5)	67 (25.1)
Around the areola	52 (15.3)	11 (15.1)	41 (15.3)
Lymph node dissection			
<10	63 (18.5)	13 (17.8)	50 (18.7)
≥10	278 (81.5)	60 (82.2)	218 (81.3)
Histopathological type			
Carcinoma simplex	212 (62.2)	53 (72.6)	159 (59.3)
Invasive ductal	86 (25.2)	13 (17.8)	73 (27.2)
Other types	43 (12.6)	7 (9.6)	36 (13.5)
ER			
Positive	199 (58.4)	33 (45.2)	166 (61.9)
Negative	96 (28.2)	29 (39.7)	67 (25.0)
Unknown	46 (13.5)	11 (15.1)	35 (13.1)
PR			
Positive	214 (62.8)	40 (50.8)	174 (64.9)
Negative	80 (23.5)	22 (30.1)	58 (21.6)
Unknown	47 (13.8)	11 (15.1)	36 (13.4)

ER, estrogen receptor; PR, progesterone receptor.

**Table II:** Association between clinicopathological factors and survival for 341 lymph node-negative breast cancer patients.

	DFS		OS	
		
	5-year, %	P-value	5-year, %	P-value
Age at diagnosis, years				
>35	85.1	0.0100^a^	95.1	0.0700
≤35	75.0		90.6	
Menopausal status				
Postmenopausal	81.6	0.2000	93.1	0.8000
Premenopausal	84.1		94.9	
Tumor diameter, cm				
≤2	86.9	0.0200^a^	94.3	0.1000
>2	78.1		93.9	
ER				
Positive	87.4	0.0060^a^	95.9	0.0009^a^
Negative	73.9		89.5	
PR				
Positive	85.5	0.1000	95.3	0.0900
Negative	77.5		92.5	

a P<0.05.

ER, estrogen receptor; PR, progesterone receptor; DFS, disease-free survival; OS, overall survival.

**Table III:** Association between hormone therapy and survival for 341 lymph node-negative breast cancer patients.

	Mean DFS, years			Mean OS, years		
		
Hormone therapy	Yes	No	P-value	Yes	No	P-value
All patients	8.3	9.3	0.003^a^	10.2	9.4	0.002^a^
Menopausal status						
Postmenopausal	9.4	8.7	0.100	10.1	9.4	0.200
Premenopausal	9.2	8.1	0.008^a^	10.1	8.5	0.006^a^
Tumor diameter, cm						
≤2	9.4	8.7	0.050	10.1	9.5	0.020^a^
>2	9.0	7.9	0.040^a^	10.1	9.0	0.030^a^
ER						
Positive	9.6	8.6	0.010^a^	10.1	9.5	0.004^a^
Negative	8.9	7.8	0.070	10.1	9.0	0.200

a P<0.05.

ER, estrogen receptor; DFS, disease-free survival; OS, overall survival.
